# GalaxyTrakr: a distributed analysis tool for public health whole genome sequence data accessible to non-bioinformaticians

**DOI:** 10.1186/s12864-021-07405-8

**Published:** 2021-02-10

**Authors:** Jayanthi Gangiredla, Hugh Rand, Daniel Benisatto, Justin Payne, Charles Strittmatter, Jimmy Sanders, William J. Wolfgang, Kevin Libuit, James B. Herrick, Melanie Prarat, Magaly Toro, Thomas Farrell, Errol Strain

**Affiliations:** 1 Center for Food Safety and Applied Nutrition, U.S. Food and Drug Administration, 20708 Laurel, MD USA; 2 Center for Food Safety and Applied Nutrition, U.S. Food and Drug Administration, 20740 College Park, MD USA; 3 DRT Strategies, 22203 Arlington, VA USA; 4 SDS Solutions, Inc, 22152 Springfield, VA USA; 5 Wadsworth Center, New York State Department of Health, NY 12201 Albany, USA; 6 Division of Consolidated Laboratory Services, Department of General Services, VA 23219 Richmond, USA; 7 Libuit Scientific LLC, 23219 Richmond, VA USA; 8 Center for Genome and Metagenome Studies, James Madison University, 22807 Harrisonburg, VA USA; 9 Animal Disease Diagnostic Laboratory, Ohio Department of Agriculture, 43068 Reynoldsburg, Ohio USA; 10 Laboratorio de Microbiología y Probióticos, Instituto de Nutrición y Tecnología de los Alimentos, Universidad de Chile, Santiago, Chile; 11 Center for Veterinary Medicine, U.S. Food and Drug Administration, MD 20708 Laurel, USA

**Keywords:** Galaxy, Biosurveillance, Whole genome sequencing, Food safety, Public health, GenomeTrakr, Genomic surveillance

## Abstract

**Background:**

Processing and analyzing whole genome sequencing (WGS) is computationally intense: a single Illumina MiSeq WGS run produces ~ 1 million 250-base-pair reads for each of 24 samples. This poses significant obstacles for smaller laboratories, or laboratories not affiliated with larger projects, which may not have dedicated bioinformatics staff or computing power to effectively use genomic data to protect public health. Building on the success of the cloud-based Galaxy bioinformatics platform (http://galaxyproject.org), already known for its user-friendliness and powerful WGS analytical tools, the Center for Food Safety and Applied Nutrition (CFSAN) at the U.S. Food and Drug Administration (FDA) created a customized ‘instance’ of the Galaxy environment, called GalaxyTrakr (https://www.galaxytrakr.org), for use by laboratory scientists performing food-safety regulatory research. The goal was to enable laboratories outside of the FDA internal network to (1) perform quality assessments of sequence data, (2) identify links between clinical isolates and positive food/environmental samples, including those at the National Center for Biotechnology Information sequence read archive (https://www.ncbi.nlm.nih.gov/sra/), and (3) explore new methodologies such as metagenomics. GalaxyTrakr hosts a variety of free and adaptable tools and provides the data storage and computing power to run the tools. These tools support coordinated analytic methods and consistent interpretation of results across laboratories. Users can create and share tools for their specific needs and use sequence data generated locally and elsewhere.

**Results:**

In its first full year (2018), GalaxyTrakr processed over 85,000 jobs and went from 25 to 250 users, representing 53 different public and state health laboratories, academic institutions, international health laboratories, and federal organizations. By mid-2020, it has grown to 600 registered users and processed over 450,000 analytical jobs. To illustrate how laboratories are making use of this resource, we describe how six institutions use GalaxyTrakr to quickly analyze and review their data. Instructions for participating in GalaxyTrakr are provided.

**Conclusions:**

GalaxyTrakr advances food safety by providing reliable and harmonized WGS analyses for public health laboratories and promoting collaboration across laboratories with differing resources. Anticipated enhancements to this resource will include workflows for additional foodborne pathogens, viruses, and parasites, as well as new tools and services.

## Background

The increasing prevalence and utility of whole genome sequencing (WGS) for pathogen genomics and metagenomics, and the retirement of older methods, like pulsed-field gel electrophoresis (PFGE), mean that laboratories need effective ways to participate in genomic surveillance and outbreak investigations. The production, analysis, and use of WGS data is a complex process that challenges many laboratories without bioinformatics support and substantial computational resources. To address this, the GalaxyTrakr system combines cloud computing with access to relevant public databases to provide a straightforward interface for scientists to run queries and obtain reliable, comparable and consistent results. A wide range of laboratories have already found this system useful. Ideally, WGS is a “democratizing force” enabling any lab around the world to use the data generated by any other lab using a WGS platform and be confident that those results are meaningful and globally accepted, without the bottleneck of a coordinating authority. This improves the speed of response to outbreaks, especially when they concern internationally traded commodities.

The amount of WGS data available for foodborne pathogens has been growing rapidly due to the efforts by U.S. public health agencies at the regional and national levels, such as PulseNet [https://www.cdc.gov/pulsenet/index.html], GenomeTrakr [1], Global Microbial Identifier (GMI) [2], the National Center for Biotechnology Information (NCBI) https://www.ncbi.nlm.nih.gov/, and their international partners, such as International Nucleotide Sequence Database (INSDC), European Molecular Biology Laboratory’s European Bioinformatics Institute (EMBL-EBI) and the DNA Databank of Japan (DDBJ) [3, 4]. Once shared, the insights derived from WGS analytics, particularly those relevant for pathogen surveillance and public health, need to be acted upon quickly and robustly.

A standard WGS run from an Illumina MiSeq sequencer at one of the public health laboratories participating in GenomeTrakr, produces ~ 1 million 250-base-pair reads for each of 24 samples. Although instrument software provides some ability to review the run and data produced, public health data must be put into context, integrating the new data and metadata sequence information obtained from any agnostic WGS source. This requires computing power and analytic software, both of which can carry significant costs [5]. Also required are trained bioinformatics personnel to interpret outputs and optimize workflows. These requirements take time, money, and shifts in priorities, but it is important that more public health laboratories become able to participate and contribute to global food safety efforts as quickly as possible.

The Center for Food Safety and Applied Nutrition (CFSAN), part of the U.S. Food and Drug Administration (FDA), saw an opportunity to build an open research capability based on the well-established, open-source Galaxy platform (https://galaxyproject.org) [6–9]. Galaxy runs in the cloud, which helps alleviate local burdens of purchasing and maintaining on-premises computation and storage hardware, including power, cooling, space, physical servers, storage arrays, etc.; cloud services also provide IT support, reducing staffing and costs further. Finally, using cloud services gives flexibility: high-memory, high-computing power, and/or flash storage can be selected to meet individual project requirements, only paying for these features as necessary.

CFSAN’s goal was to enable laboratories outside of the internal FDA network to perform quality assessments of sequence data and identify links between clinical isolates and positive food/environmental samples in the NCBI SRA (https://www.ncbi.nlm.nih.gov/sra/). In contrast to commercialized platforms, this new resource, called GalaxyTrakr (https://www.galaxytrakr.org), would eliminate the need for individual laboratories to purchase yearly subscriptions to commercial bioinformatics packages and enable consistent and harmonized interpretations of WGS results across laboratories by providing tools optimized for food pathogen surveillance. While some of these curated tools originate in the public Galaxy Toolshed (https://toolshed.g2.bx.psu.edu), GalaxyTrakr hosts additional tools to support GenomeTrakr and other public health applications. As more applications are developed, this toolset will expand. These tools can be further customized by participants to meet local needs, and those changes can be rapidly shared with other GalaxyTrakr users, rather than waiting for an external company to release a new version of software.

**Fig. 1:**
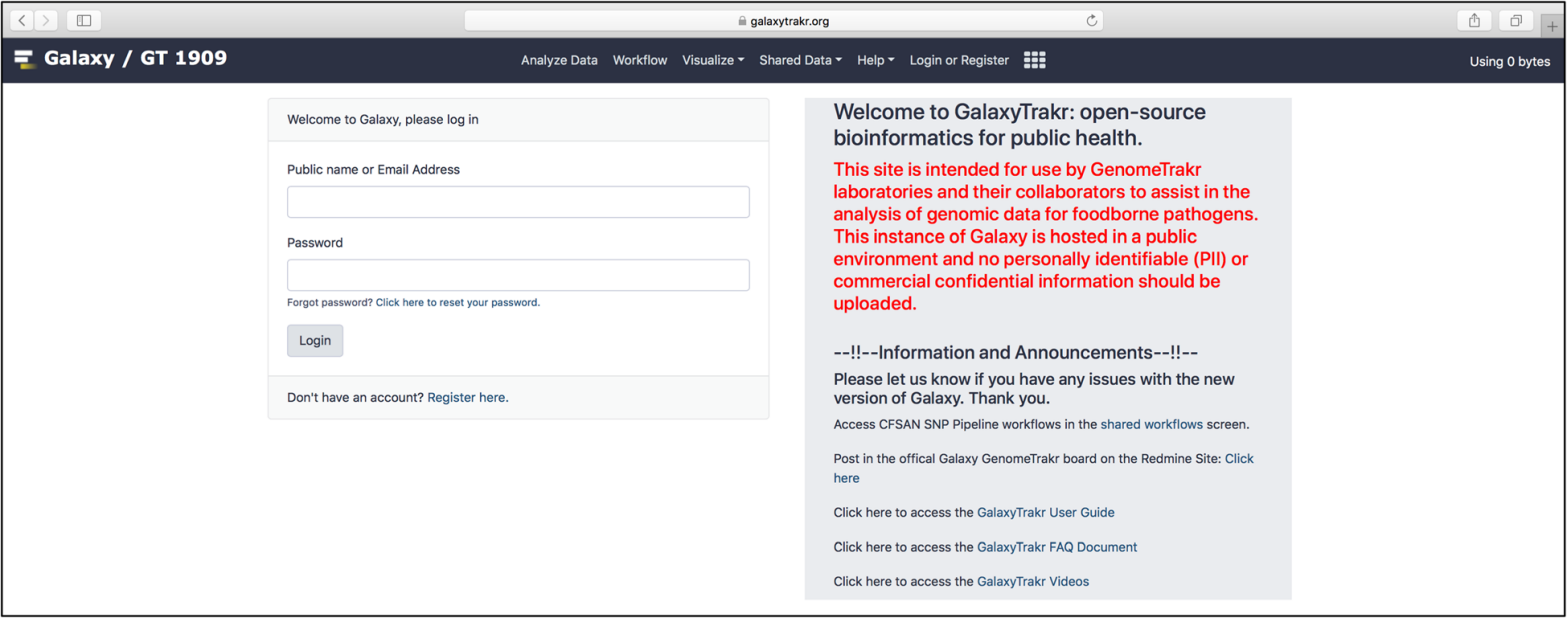
GalaxyTrakr login page where user can enter their login information and browse through documents, such as a user manual

**Fig. 2:**
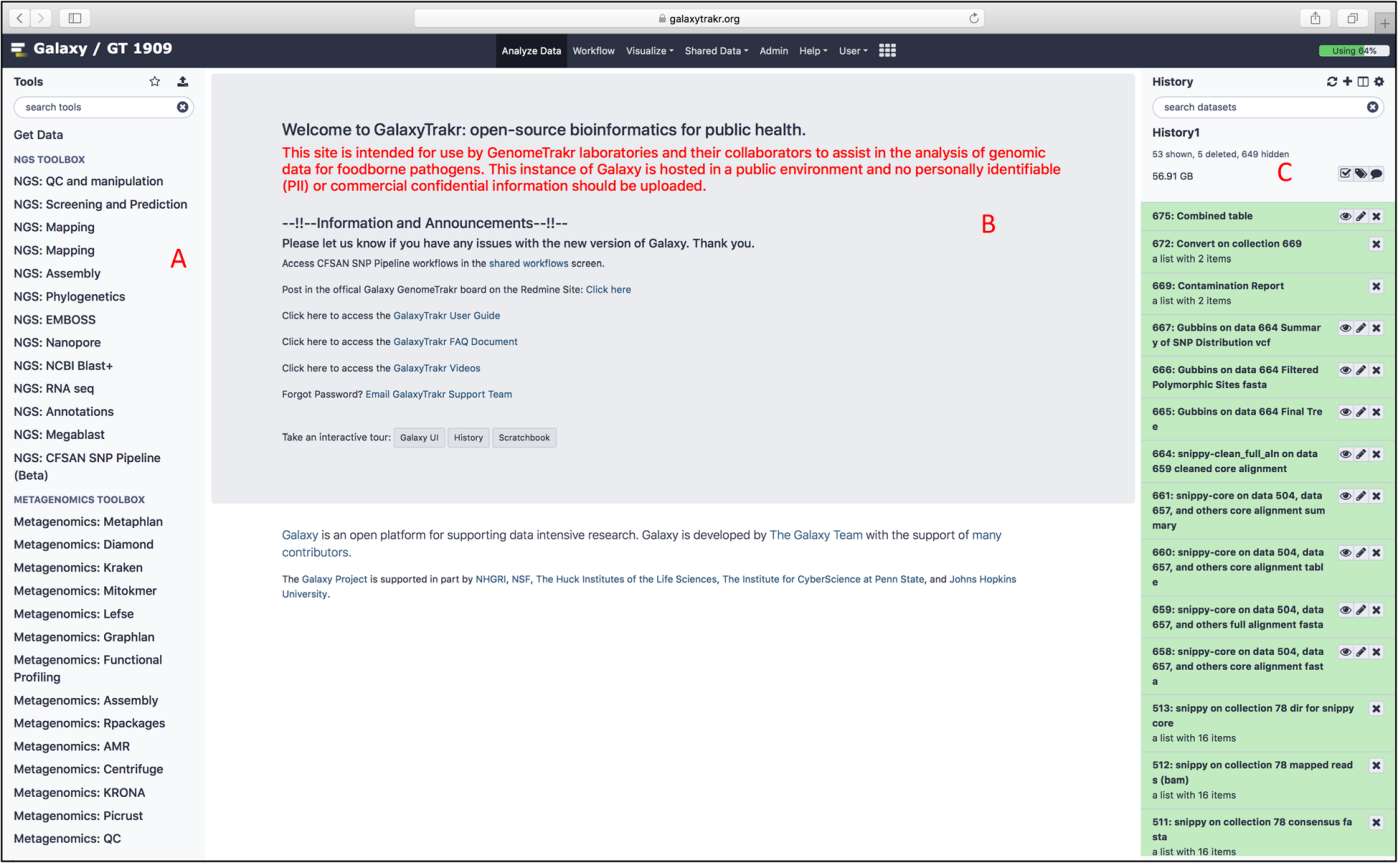
GalaxyTrakr user interface that contains three panels: **a** Available tools, **b** Data analysis, and **c** File histories

By providing tools and workflows for analysis of foodborne bacterial pathogens, GalaxyTrakr promotes collaboration between federal, state, and academic institutions, and provides ready access to bioinformatic tools for non-bioinformaticians in public health (Table 1). Laboratories can now share data, insights, and quality control (QC) standards across state, federal, and international boundaries which allows more timely intervention to support public health. This work will also spur more rapid uptake of WGS in other types of disease surveillance. Once logged into the GalaxyTrakr environment (Figs. 1 and 2), users can perform a comprehensive end-to-end workflow. A basic workflow might be as follows: (1) import sequence data from local data sources (e.g. the investigator’s own sequencer) or from NCBI SRA, (2) perform quality control and trimming, (3) run *de-novo* assembly using one of several high-performance de Brujin-graph assemblers [10, 11], (4) serotype, multiple locus sequence type (MLST) and/or predict antimicrobial resistance using tools such as SeqSero [12] and ABRicate (https://github.com/tseemann/abricate), (5) perform phylogenetic analysis using the CFSAN SNP Pipeline [13] and RAxML [14], and (6) create dendrogram visualizations using the Newick Display [15]. In addition to these tools, GalaxyTrakr also provides small custom tools that will collate and summarize individual results of workflows. More advanced users can customize the provided workflows and build additional ones tailored to their needs.

**Table 1:** The most commonly used tools in GalaxyTrakr based on the number of jobs run over the years 2017–2020

Tool ID	Use	No. of jobs run
FastQC	NGS Quality assessment	39750
SNP_pipeline^a^	SNP identification	36685
Trimmomatic	NGS Quality assessment	25138
SkesaMLST	Short-read assembly and MLST Typing	17641
SPAdes	Short-read assembly	13962
SeqSero	*Salmonella* Serotyping	10788
Fastq_dump_paired	Paired Fastq reads download	9514
ABRicate	Antimicrobial Resistance	8520
AMRFinder	Antimicrobial Resistance	6976
SKESA	Short-read assembly	5791
Ectyper	*E. coli* serotyping	5237
QUAST	Quality assessment	4475
Kraken2	Metagenomics classification	4433
shovill	Short-read assembly	3760
MetaPhlan2	Metagenomics classification	2685
RAxML	Phylogenetics	2649
Mlst	MLST Typing	1711
Prokka	Genome annotations	1382
Confindr	Bacterial contamination detection	1110
Btyper	Classification of *Bacillus cereus* group	1077
SRST2	Short-read sequence typing	1050
Mitokmer^a^	Metagenomics eukaryotic prediction	975
Sistr_cmd	*in silico* serotyping	933
Metaspades	Metagenomics assembly	725
Newick Display	Phylogenetics display	720
Bwa_mem	Short-read mapping	637

^a^Tools that are specifically designed for GalaxyTrakr users

## Implementation

The GalaxyTrakr team consists of a staff of personnel with expertise in the area of cloud engineering, software development, bioinformatics, and general information technology system administration. In order to create GalaxyTrakr, a team of six people was involved in developing the requirements, designing the architecture and identifying the curated tools that should be included in support of food safety. The deployment of the necessary resources required one cloud engineer and one bioinformatician. To provide ongoing operations and maintenance of the GalaxyTrakr platform, the team consists of ten people that also support other platforms for CFSAN. During the design phase, it was determined that the team would leverage the Galaxy bioinformatics platform to provide the web based graphical user interface that could host the bioinformatics tools, thereby enabling collaboration among multiple agencies. In addition to the Galaxy platform, it was necessary to design a scalable computing infrastructure to provide the processing power for the tools that would be utilized within the GalaxyTrakr platform. The team decided to leverage the scalable computing options offered by the Amazon Web Services (AWS) cloud service provider. One of the major benefits of using a cloud service like AWS to host the Galaxy application is that it is a “pay-as-you-go” service. This is beneficial because our use-case is generally episodic in nature and so this is typically less expensive than making an investment into on-premise computing and storage hardware. In addition, a cloud service also provides IT support for the services being used, allowing the GalaxyTrakr team to focus on the application software and infrastructure. More importantly, a cloud service provider eliminates the need to provide support for resources that would be necessary to host the application on-premise, such as power, cooling, space, physical servers, storage arrays, etc. This reduces staffing level of effort and cost. Lastly, cloud service gives the flexibility to use various computing options, such as high-memory, high-compute, or flash storage to meet individual processing requirements, and at the benefit of only paying for it when it is needed.

The primary requirement for getting approval to use the GalaxyTrakr platform is to be a part of or support a food safety program. The process required to gain access to the system is straightforward. Using any web browser of choice, navigate to https://account.galaxytrakr.org/account/register to complete a user registration form. When filling out the registration form, please be as complete as possible with the requirements section; this section is useful in evaluating user’s application and for our annual funding request. Once the form is submitted, the request will be evaluated by the GalaxyTrakr team and the request will be either approved or denied. If the request is approved, a notification will be sent to the user with the credentials for GalaxyTrakr along with links to the user guide. The user guide outlines key instructions on accessing and utilizing the platform. In order to use GalaxyTrakr, a user simply needs to have a personal computer that is connected to the public internet and has either the Chrome or Firefox web browser. By default, a user will be allowed to use up to 250 GB of storage space within their GalaxyTrakr histories. The storage quota can be increased by reaching out to the GalaxyTrakr support team (galaxytrakrsupport@fda.hhs.gov). Currently, there is no limit set on processing units utilized by an end user. For those desiring an independent instance of Galaxy, the Galaxy platform provides documentation on how to deploy and utilize the application in various scenarios. It is not necessary to run Galaxy within a cluster computing environment. Users can deploy Galaxy on a stand-alone system for testing or developing tools or to perform a small amount of processing. When first starting with Galaxy, we recommend deployment on a stand-alone system. The initial deployment documentation can be found at: https://galaxyproject.org/admin/get-galaxy/. However, if the Galaxy platform will be utilized by more than five people at the same time, it is recommended to configure Galaxy so that the tasks are run within a cluster computing environment. Utilizing a cloud service like AWS is a great option if an existing computing cluster is not available. Galaxy provides a “packaged” cluster computing environment that leverages AWS via a service called CloudMan [16]. A typical deployment of CloudMan can be completed in less than an hour and documentation on the steps required are available (https://galaxyproject.org/cloudman/getting-started/). If deploying for use by a large community or an enterprise, it is recommended to deploy in a purpose-built cluster computing environment. It is straight-forward to do a basic cluster computing setup using Cloud Formation Cluster (CfnCluster). Deployment instructions for CfnCluster are available at https://cfncluster.readthedocs.io/en/latest/. Once the cluster is deployed, the Galaxy software can be installed on the master node and configured to leverage the clustered environment. Tools developed and maintained by the GalaxyTrakr team can be installed out of the GalaxyTrakr tool repository (https://toolrepo.galaxytrakr.org/).

## Results

**Fig. 3:**
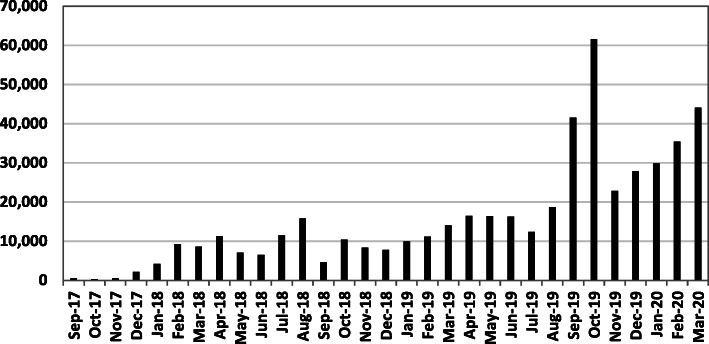
Number of monthly jobs run on GalaxyTrakr through March 2020

A wide range of laboratories have already found GalaxyTrakr useful, ranging from public health laboratories to veterinary and academic laboratories. In its first year, GalaxyTrakr processed over 85,000 jobs and went from 25 to 250 users, representing 53 different public and state health laboratories, academic institutions, international health laboratories, and federal organizations, growing to 600 registered users from 87 labs (Table 2) and 450,000 analysis jobs by March 2020 (Figure 3)**.** While many of the jobs run in GalaxyTrakr are responding to current public health issues, others are in support of regulatory and research topics which may guide future public health decisions. Several laboratories not only use GalaxyTrakr for their own analyses and data-sharing, but also share their expertise with other laboratories and academic partners, which continually expands the scope of GalaxyTrakr’s contributions to public health. To demonstrate how GalaxyTrakr can be used to bring powerful genomic tools to a wider range of users and train the next generation of genomic scientists, we present the following case studies, followed by instructions for laboratories interested in joining the GalaxyTrakr project.

**Table 2:** GalaxyTrakr user information from various national and regional organizations

Organization type	Organization level	No. of organizations	No. of users	Geographical region
human and animal health	federal	5	186	North America
agriculture	federal	1	7	North America
human and animal health	state	49	209	North America
agriculture	state	4	8	North America
education and research	not applicable	17	200	North America
human and animal health	federal	1	3	Asia
human and animal health	federal	2	4	Europe
education and research	not applicable	1	7	Europe
education and research	not applicable	2	2	Africa
human and animal health	federal	1	1	South America
education and research	state	1	1	South America
company	not applicable	3	7	not applicable
Total		87	635	

### Laboratory profiles

#### Wadsworth Center, New York State Department of Health

The team at Wadsworth has successfully run over 26,000 jobs through GalaxyTrakr, primarily to confirm *Salmonella* serotypes using the SeqSero tool and more recently MicroRunQC (a CFSAN workflow) for quality assurance and quality control. As this lab is also part of PulseNet, Wadsworth provides a useful illustration of how GalaxyTrakr can bridge the past (i.e., PFGE-based identification of outbreaks, which was retired by CDC in 2019) and the future of pathogen surveillance. The Wadsworth Center was one of the laboratories working on both platforms, completing several analyses of PulseNet organism outbreaks using the GalaxyTrakr pipeline to support findings from both an in-house pipeline [17] and the NCBI Pathogen Detection Browser (https://www.ncbi.nlm.nih.gov/pathogens/isolates#/search/). Going forward, the Wadsworth Center will continue to use GalaxyTrakr to perform QA/QC on GenomeTrakr isolates, WGS phylogenetic analysis of outbreaks for which the cgMLST scheme in BioNumerics (supported by PulseNet) is not available (such as *Clostridium perfringens*) or in cases where cgMLST may not provide adequate resolution (i.e. *Salmonella* Enteritidis). By using the validated CFSAN SNP Pipeline in GalaxyTrakr, Wadsworth scientists will be able to provide reliable SNP cluster analyses to support local outbreak investigations. GalaxyTrakr has also served as a valuable tool to complete some retrospective analyses of outbreaks of non-PulseNet organisms, including *Clostridium perfringens* [18]. The Wadsworth lab has developed their own tools and pipelines, which can be used for local analyses as well as assessing and improving shared resources. One ongoing study is comparing phylogenetic analyses of *Salmonella* and *E. coli* samples performed using their in-house Wadsworth Center pipeline, NCBI_PD and the CFSAN SNP pipeline. While preliminary results indicate a strong concordance between these three pipelines, a few clusters exhibited noticeably different SNP diversity (data not shown). Such results can help researchers determine which cases may be sensitive to pipeline assumptions and would therefore require manual review.

#### Ohio Department of Agriculture/Animal Disease Diagnostic Laboratory (ADDL).

 ADDL provides veterinarians with a wide variety of diagnostics for food animals, horses, companion animals, and exotic species, performing over 30,000 molecular diagnostic tests per year (https://agri.ohio.gov/wps/portal/gov/oda/programs/animal-disease-diagnostic-lab/lab-sections). Their work includes surveillance for reportable animal diseases as well as illnesses caused by contaminated animal feed, including pet foods (https://www.fda.gov/animal-veterinary/science-research/veterinary-laboratory-investigation-and-response-network). The ADDL team began using GalaxyTrakr in 2017 and now uses GalaxyTrakr for all client bacterial WGS data analysis, in addition to analyses sent on to GenomeTrakr and the Veterinary Laboratory Investigation and Response Network (Vet-LIRN), an FDA surveillance network for animal pathogens and feed contamination. Prior to having GalaxyTrakr access, ADDL relied on classic microbiological methods, such as culture-based methods to determine antibiotic susceptibility and *Salmonella* serotypes, that often took days to complete. In contrast, GalaxyTrakr analyses allows ADDL to use genomic techniques to immediately perform assemblies and characterize isolates: once a sequencing run is completed, they upload their FASTQ files into GalaxyTrakr via file transfer protocol (FTP), perform quality control checks, followed by assembly and analysis. Convenient workflows allow ADDL scientists to run multiple tools together, or sequentially, depending on need. Completed genome assemblies and tabulated data outputs (e.g., a table of *Salmonella* serotypes) can then be downloaded from GalaxyTrakr to a local storage drive. These data outputs, such as predicted antibiotic resistance genotype, serotype, and multilocus sequence type, can be easily organized into reports to be shared with stakeholders. Those reports are stored in local ADDL databases, ready to be used for future temporal and diagnostic studies. Being able to perform rapid independent analyses of isolates was especially beneficial during the multistate *Campylobacter jejuni* outbreak in 2017–2018 that was linked to puppy exposure [19]. Having continuous access to GalaxyTrakr tools enabled ADDL to quickly identify multi-drug resistant *C. jejuni* collected from animals, then share that information with colleagues at the Ohio Department of Health and CDC who were investigating the human-related illnesses attributed to the outbreak. Another use for GalaxyTrakr tools is generating data suitable for FDA analysis packages, which are necessary for regulatory actions. In 2018, ADDL worked with the Ohio Department of Agriculture Consumer Protection Laboratory to provide bacteria isolation, identification, and WGS in response to microbial adulteration of two commercial pet foods linked to pet and/or human illnesses that led to recalls (https://wayback.archive-it.org/7993/20180126102242/https://www.fda.gov/Safety/Recalls/ucm508394.htm, https://www.odh.ohio.gov/wps/portal/gov/odh/know-our-programs/food-safety-program/food-recalls/03212018-radagast-pet%20food-cat-food, https://www.odh.ohio.gov/wps/portal/gov/odh/know-our-programs/food-safety-program/food-recalls/g-and-c-raw-pats-cat). Harmonized, shareable workflows hosted on GalaxyTrakr made inter-laboratory analyses simple and consistent. Looking ahead, ADDL plans to develop workflows which could be used by the entire Vet-LIRN WGS laboratory network, helping to support human and animal health.

#### Virginia Division of Consolidated Laboratory Services (DCLS) 

GalaxyTrakr has become a critically important bioinformatics hub for state public health laboratories in the United States. DCLS, Virginia’s public health laboratory, has been an active user of GalaxyTrakr since the project’s launch. DCLS scientists have also used Galaxy and GalaxyTrakr to train public health laboratory scientists in applied microbial genomics to ensure laboratories without bioinformatics specialists can quickly and reliably analyze WGS data. In 2018, DCLS partnered with the Applied Bioinformatics Laboratory (ABiL) to develop an introductory bioinformatics workshop for public health scientists in the Mid-Atlantic region. To provide an engaging hands-on educational experience, the Galaxy interface was selected as the primary application for course content. Trainees were taught to navigate Galaxy’s web interface and to perform WGS analyses using the various Galaxy plugins. Instructors delivered critical background information on common analytical approaches for assessing read quality, performing genome assembly, and mapping read data all while participants performed the analyses themselves. By using Galaxy, DCLS has been able to help public health scientists with molecular and microbiology backgrounds better understand bioinformatics principles and learn how to interpret results, without the additional complexity of mastering a command-line interface and/or computer science jargon, both of which can be barriers to participating in genomics work. In a post-training survey, 100 % of participants reported that they planned to apply the skills and knowledge they learned from this training to their work in public health. Trainees in this workshop were able to register for GalaxyTrakr.org access upon completing the program, regardless of their affiliation to the FDA’s GenomeTrakr program. This transition from Galaxy to GalaxyTrakr has been helpful for laboratories seeking Galaxy plugins specific to public health investigations, e.g., the CFSAN SNP pipeline. State laboratories across Virginia also benefit from having access to the dedicated Amazon Web Services (AWS) computational resources, active FDA user-support, and training material made available by the GalaxyTrakr team.

#### State Public Health Bioinformatics (StaPH-B)

As word about GalaxyTrakr has spread, additional state public health laboratories who are not part of the GenomeTrakr program have requested and been granted access. In part, promotion of GalaxyTrakr has been through the State Public Health Bioinformatics (StaPH-B) workgroup. StaPH-B is a consortium of public health scientists actively addressing the common barriers impeding the implementation and use of bioinformatics in state laboratories. StaPH-B consists of roughly 60 scientists from 25 public health laboratories, and has been helping promote GalaxyTrakr as part of their mission to reduce barriers to using bioinformatics in state laboratories. Many StaPH-B laboratories are not affiliated with the FDA’s GenomeTrakr project. However, in June 2019, through a webinar presentation, GalaxyTrakr staff introduced GalaxyTrakr to StaPH-B using a well-received webinar and made the platform available to the entire workgroup. GalaxyTrakr has since been adopted by several StaPH-B laboratories and has become a valuable resource for the state public health community.

#### James Madison University

 The Center for Genome and Metagenome Studies (CGEMS) at James Madison University (JMU) (https://www.jmu.edu/genomics/about.shtml) has been working with DCLS as part of a collaborative Course-based Undergraduate Research Experience (CURE) project to employ GalaxyTrakr in an undergraduate advanced microbiology course. Students in BIO 346 *Bacterial Discovery* study the genomics of *Salmonella enterica* isolated from environmental sources. In this required course, advanced microbiology students first learn how to isolate and identify *Salmonella* obtained from local stream sediments and poultry litter. After using standard microbiology techniques and PCR of the genus-specific *invA* locus to isolate and positively identify their strains, the genomes of their isolates are sequenced by DCLS. DCLS provides their Illumina reads of JMU *Salmonella* isolates directly to a GalaxyTrakr data library, which the students can access. Students learn to use basic tools on GalaxyTrakr such as FastQC [20], SPAdes, and QUAST [21] for *de novo* genome assembly and quality control and learn to annotate their genomes using Prokka [22]. *In silico* serotyping using SeqSero and annotation of antibiotic resistance genes using ABRicate can also be performed on GalaxyTrakr. Further analyses using web-based tools can then identify potential plasmids and other mobile elements (MEs) such as genomic islands, transposons, integrons, etc. All these data are combined with wet lab phenotypic data to confirm antibiotic susceptibility and the presence of MEs. Students also have the opportunity to sequence a subset of interesting isolates using the ONT MinION (Oxford Nanopore Technologies, Oxford, UK, https://nanoporetech.com/) and perform hybrid-assembly of the data. During the piloting and launch of the course in 2018, JMU undergraduates isolated 56 unique strains of *Salmonella*, with 5 from poultry litter and 51 from stream sediments. Fifty-two isolates have been sequenced to date and, of these, 17 *Salmonella* serotypes have been identified, including the potential human pathogens *S.* Typhimurium, *S.* Braenderup, *S.* Montevideo, and *S.* Infantis. Two of the isolates were shown to carry a plasmid-borne gene encoding resistance to fluoroquinolones (qnr) such as ciprofloxacin. One of the strains, an *S.* Infantis isolated during the pilot of the course in the spring, was found to harbor a very large (300 kb) plasmid encoding resistance to multiple antibiotics and metals. Twelve students took the course in the spring and eleven in the fall semester of 2018. In addition to teaching students at JMU, CGEMS has provided bioinformatics training to other groups. In July 2018, an abbreviated introduction to the bioinformatics portion of Bacterial Discovery was given to approximately 30 faculty and students from Virginia in a 3-day workshop sponsored by CGEMS. This workshop and the spring semester of the BIO-346 course have generated data for two Microbiology Research Announcements and BIO-346 was the subject of an honors thesis. Based on what we have learned, a pair of teaching modules, one in pathogen microbiology and the other in genomics, are being written up for publication. Because of the CGEMS workshop, Longwood University’s Department of Biology and Environmental Sciences (Longwood, VA, USA) is now piloting a new genomics course that will also use GalaxyTrakr to study *Salmonella*.

#### Laboratorio de Microbiología y Probióticos, Instituto de Nutrición y Tecnología de los Alimentos (INTA), Universidad de Chile, Santiago, Chile

Since GalaxyTrakr is a cloud-based system, laboratories from around the world can participate, which is essential for protecting the global food supply. The Laboratorio de Microbiología y Probióticos at the Instituto de Nutricion y Tecnología de los Alimentos (INTA), Universidad de Chile, has collaborated with CFSAN and submitted isolates to be archived in GenomeTrakr since 2015. The primary goal at INTA has been to characterize foodborne pathogens in Chile and understand the underlying diversity within species. Dozens of Shiga toxin-producing *Escherichia coli* (STEC) and *Salmonella* isolates have already been sequenced and analyzed, and ~ 600 more *Salmonella* from surface waters will be sequenced during 2019/2020. Initially, Chilean isolates were sent for sequencing and analysis in the U.S., which was cumbersome and slow. While isolates are still being sequenced in the U.S., using GalaxyTrakr allowed researchers at our laboratory to have convenient and free access to powerful tools for accessing that sequence data and analyzing those data locally. Such free tools are very important because research funding for purchasing bioinformatics software is scarce in Chile. GalaxyTrakr allowed users without a strong bioinformatics background to analyze data and quickly share results with international partners. The current INTA workflow for *Salmonella* isolates, collected in Chile and sequenced in the U.S., starts with downloading the data (in Chile) using the GalaxyTrakr tool FASTQ Dump paired downloader from the SRA-toolkit http://ncbi.github.io/sra-tools/, followed by quality processing with FASTQC, and Trimmomatic [23]. Genomes are assembled with SPAdes and Shovill (https://github.com/tseemann/shovill), characterized to determine serotype, antimicrobial resistance genes, and multilocus sequence type [24] (https://pubmlst.org/mlst/), and then annotated. Finally, RaXML is run to reconstruct the most likely phylogenetic relationships among isolates, allowing identification of clusters. These reconstructions let users assess the diversity of Chilean *Salmonella*. Researchers at our laboratory have also used GalaxyTrakr to perform phylogenetic analyses of STEC isolates, thereby identifying a wide diversity in Chilean STEC and their relationships (unpublished data). By enabling users to run independent analyses without needing trained bioinformaticians and expensive investments in bioinformatics software or computational resources, GalaxyTrakr has become a very valuable tool. Participating in GalaxyTrakr has also helped build our laboratory’s workforce: undergraduate and graduate biology and veterinary students without extensive bioinformatics knowledge can now contribute to genomics research, thereby greatly increasing productivity. Having access to these tools also helps the team prepare scientific manuscripts documenting the prevalence and characteristics of environmental foodborne pathogens in Chile. These insights will support future advances in global food safety and pathogen surveillance.

## Discussion

As is increasingly clear, outbreaks of foodborne illness need to be identified and managed as quickly as possible. Creating and maintaining effective pathogen surveillance is essential to global public health. GalaxyTrakr facilitates surveillance in several ways. It increases the number of laboratories able to participate in surveillance and outbreak investigations, helping even those with limited budgets analyze their data by removing expense and expertise barriers. Laboratories can customize tools from the toolshed and share those tools across organizational and national boundaries, which is critical when the food supply is global in scope. Key to the impact of WGS bio-surveillance on public health is the rapid application of its insights; anything that reduces the time from sample acquisition to actionable insight reduces the economic and medical impact of an outbreak of foodborne disease. GalaxyTrakr enables WGS data to be analyzed almost directly off the sequencer by local laboratories which could be in the best position to intervene in an outbreak often hours or days before that data becomes public in the FDA GenomeTrakr project and the NCBI Pathogen Detection Portal. Being able to make these contributions provides a better sense of local ownership in the process, improves cooperation, and takes advantage of local knowledge – an important win, since reliable shared insights allow faster interventions to protect public health.

Other instances of Galaxy are supported by various organizations and can also be used to address food safety questions in public health. The instance of Galaxy most similar to GalaxyTrakr is the Advanced Research Infrastructure for Experimentation in genomicS (ARIES) [25]. The GalaxyTrakr team has worked directly with the ARIES team to share tools between the platforms. More generally, teams share tools using the Galaxy Toolshed, which provides a sharing mechanism across all Galaxy instances. The availability of these different instances of Galaxy means that users can choose the one that best suits their needs, and if one site is down or loses support, another site can be used.

There are numerous software packages that compete with Galaxy. Generally, these packages focus on specific topics and do not span the breadth of tools available in the Galaxy toolshed. The most mature Reproducible Research System (RRS) platforms competing with Galaxy in the realm of microbial genomics are GenePattern [26] and Mobyle [27, 28]. The advantages of Galaxy over other alternatives are, in addition to the freely available tool resource, Galaxy allows users to design and execute custom workflows, giving some users command of complex pipelines, and provides the ability to remix analyses to suit their own needs. Commercial offerings such as those produced by CLCbio such as genomic workbench (https://digitalinsights.qiagen.com) are available, but these are prohibitively expensive for global access, as well as being difficult to customize or add to.

Genomics offers amazing opportunities to understand pathogens at a molecular level, including antimicrobial resistance (AMR), virulence potentials, and culture-independent analyses. These tools can be applied not only to human pathogens, but also to veterinary and food crop pathogens and to investigate microbiomes. Work is already underway to include workflows for additional foodborne pathogens, viruses, and parasites, as well as new tools and services for both genomic and metagenomic analyses. In addition to this, genomic tools provide opportunities to understand pathogen movement through global commerce and travel.

The Public Health Alliance for Genomic Epidemiology has provided ten recommendations for supporting open pathogen genomic analysis in public health settings [29]. In taking advantage of Galaxy to produce the GalaxyTrakr platform, we have addressed four of these: (1) bioinformatic pipelines are open-source and broadly-accessible, (2) pipelines provide for data visualization, exploration, and automated analysis by non-bioinformaticians, (3) results can be shared and reproduced, and (4) cloud computing allows for a scalable system with wide accessibility. Meeting these recommendations has allowed us to provide a strong platform to support public health efforts based on WGS.

## Conclusions

We started the GalaxyTrakr project with both a problem and a question in mind. The problem is one facing public health in general – how to best ensure public health in a world with a high rate of movement of goods, people, and associated diseases. The genomics and sequencing revolution of the last 30 years has the potential to be part of the solution to this problem, and lead to our question: how can we effectively share the benefits and potential of the genomic revolution for foodborne pathogens across all public health laboratories, including those without bioinformatics staff, in a way that fosters mutual cooperation? The solution requires that WGS data from foodborne pathogens be widely available. Fortunately, the GenomeTrakr network has addressed this need by centralizing data deposition at the NCBI/EMBL/DDBJ and encouraging all food safety organizations to submit data. With the data readily available to all, the answer focuses on providing tools that allow analysis and processing of WGS data, both data generated locally, and all publicly available data.

We took full advantage of an open source platform (Galaxy) and many of the public tools available in it. Combined with a modern cloud-computing infrastructure, this has allowed us to support a substantial number of public health efforts in a cost-effective manner. Additionally, the groundwork we have laid in foodborne disease response will readily support efforts in other diseases. The uptake of GalaxyTrakr so far indicates that a powerful set of tools can be arranged in an environment that novices can join and use effectively. CFSAN is pleased with the success of this initiative and intends full support of the GalaxyTrakr platform for the foreseeable future. Stakeholders have been impressed by the relatively modest cost for the results and are encouraging us to continue to promote and grow the system. Organizations and individuals that work in public health are welcome to request accounts and participate in the project to the extent that makes sense for their needs.

## Availability and requirements

**Project Name:** GalaxyTrakr.

**Project Website:** https://galaxytrakr.org

**Operating system(s):**UNIX/Linux or MacOS (Galaxy); Platform independent for Galaxy’s browser-based user interface.

**Programming language:** Python 3.5+

**Other requirements:** Amazon Web Services (AWS) Commercial U.S. East-1, AWS ParallelCluster 2.6, Centos 7, Galaxy 19.09, SGE 8.1.9, PostGreSQL 9.6, nginx 1.12

**License:** Galaxy source code is licensed under the Academic Free License version 3.0.

**Any restrictions to use by non-academics:** None.

## Data Availability

The user guide outlines key instructions on accessing and utilizing the GalaxyTrakr platform can be found in the following link.https://s3.amazonaws.com/cfsan-genometrakr-docs/user+guide/Galaxy+Genome+Trakr+User+Guide.pdf
